# Early Surgery and Screw-Only Osteosyntheses in Minimally Invasive Treatment of Calcaneal Fractures—Risk or Benefit for Our Patients?

**DOI:** 10.3390/jcm14020344

**Published:** 2025-01-08

**Authors:** Christian Rodemund, Moritz Katzensteiner, Maximilian Vogel, Georg Mattiassich

**Affiliations:** 1 Independent Researcher, Alpenblickstrasse 38, 4060 Leonding, Austria; 2 Klinik Diakonissen Schladming, Salzburger Straße 777, 8970 Schladming, Austria; moritz.katzensteiner@outlook.com; 3 Trauma Center Linz, Garnisonstrasse 10, 4060 Linz, Austria; maximilian.vogel@auva.at (M.V.); georg.mattiassich@auva.at (G.M.)

**Keywords:** minimally invasive surgery, intra-articular calcaneal fractures, screw-only osteosynthesis, surgical timing, percutaneous surgical technique, sinus tarsi approach

## Abstract

**Background:** This study aims to analyze the outcomes following the minimally invasive surgery of calcaneal fractures using screw-only osteosynthesis, as well as the impact of surgical timing. **Methods:** Between 2015 and 2020, 155 patients with 168 fractures were included. According to the Sanders classification, 48.21% of fractures were classified as Sanders 2, 33.33% as Sanders 3, and 10.11% as Sanders 4 fractures, with the remaining fractures unclassified. A total of 117 cases were treated on the day of admission or the following day. The surgeries followed a standardized protocol for fracture analysis, positioning, and X-ray techniques, primarily using a percutaneous approach with stab incisions. Osteosynthesis was mainly performed using screws, with five cases treated with K-wires for open fractures. **Results:** The mean Boehler’s angle improved from 8.52° preoperatively to 25.00° postoperatively. Three superficial infections were observed. Deep infections occurred in three cases, all following open fractures. Secondary dislocation was noted in five patients—one due to a deep infection, and four attributed to clear technical failures. One case involved a questionable indication for a screw change (7.3 mm screw) after two weeks due to perforation of the medial wall. A total of 79 fractures were followed up for an average of 4 years and 3 months. The mean AOFAS score was 91.3, and the mean FAOS score was 88.7. Surgery within 7 days after admission showed no significant impact on the outcomes. **Conclusions:** Minimally invasive screw-only osteosynthesis with early surgical intervention offers favorable outcomes with minimal risk.

## 1. Introduction

Advancements in diagnostic means, innovative techniques, and specialized implants have significantly enhanced surgical treatment options for calcaneal fractures and largely replaced conservative therapies [1,2,3,4,5]. One of the key developments was the introduction of the extended lateral approach combined with plate osteosynthesis, which emerged as the ‘gold standard’ at the turn of the millennium, although it is associated with a risk of soft-tissue complications [6,7,8].

Due to this disadvantage, minimally invasive treatment strategies have gained attention as promising alternatives [9,10]. The most well-established technique features the sinus tarsi approach and osteosynthesis with specialized plates [11,12,13,14,15]. This method notably reduces complication rates and achieves favorable reduction and clinical outcomes, making it ideal for moderate fractures patterns (Sanders types 2 and 3). However, in the case of comminuted fractures, it does not provide the same level of visualization to the fracture situation and has limited options for reduction compared with the extended lateral approach [16].

In addition, percutaneous procedures utilizing only stab incisions and no plates have gained popularity because of their very low complication rates and the possibility of immediate surgical intervention [17,18,19]. Some argue that achieving adequate reduction is challenging, and osteosynthesis with screws alone may lack the stability required to prevent secondary dislocation or to facilitate early fixation-free rehabilitation, but in our study, we could not confirm these concerns.

The evolution of surgical techniques has also changed time management. While waiting periods of 7 to 14 days are typically advised for the extended lateral approach [20], a reduced time of 4 to 6 days for the sinus tarsi approach is common [21,22], with recommendation to intervene even earlier for percutaneous techniques [9,23].

Our study focuses specifically on minimally invasive treatment techniques and screw-only osteosynthesis due to its distinct advantages in reducing soft-tissue complications and facilitating early surgical intervention. This technique eliminates the need for large incisions or bulky hardware, which are often associated with postoperative wound complications. Our results, in conjunction with a review of the literature, determine if this technique can provide feasible outcomes compared to the ELA and the sinus tarsi approach and if it allows for stabilization equivalent to established methods, such as plate osteosynthesis, to enable secure bony healing and early functional rehabilitation, which is increasingly recognized as crucial for optimizing clinical outcomes.

Another key focus of our study is the impact of the timing of surgery after admission on clinical outcomes—an area that remains underexplored in the context of minimally invasive techniques combined with screw-only osteosynthesis.

## 2. Materials and Methods

### 2.1. Data Base

The data for this retrospective study were collected at a local trauma center of the Workers Compensation Board Austria (AUVA) in Linz, Austria, (Trauma Center Level 2). The inclusion criteria encompassed all patients with surgically managed calcaneal fractures between 2015 and 2020.

The demographic data (age, sex, and comorbidities,) and clinical parameters (fracture classification, surgical techniques used, and length of hospital stay, etc.) were collected.

We recorded the time interval between injury and hospital admission at our facility, as well as the date and time of surgery, to evaluate the timing of surgical intervention. Postoperative complications, such as wound healing disturbances, infections, or the need for secondary surgical interventions, were systematically extracted from the electronic medical records.

All data were anonymized and stored in a secure database.

In the analyzed period, 155 patients with 168 surgically managed injuries were treated. Including 2 reoperations, a total of 170 minimally invasive surgical procedures were performed.

The fractures were grouped according to the Sanders [24] and Essex-Lopresti [25] classifications. The majority of cases were Sanders’s type 2 (81 cases, 48.21%), followed by Sanders’s type 3 (56 cases, 33.33%). According to the Essex-Lopresti system, there tended to be more depression-type fractures (87 cases, 51.78%) and fewer tongue-type fractures (56 cases, 33.33%).

Open fractures were observed in 15 cases. Two of these patients had no additional injuries. In all other cases, severe fractures to the spine, pelvis, chest, and extremities were detected. According to the Sanders classification, there were two type 2A fractures, one type 3AB fracture, eight type 4 fractures, and four fractures that could not be classified (Table 1).

A total of 10 patients sustained polytraumas, all resulting from falls from height and all with severe calcaneal fractures (12 fractures: 3 Sanders type 3AB, 9 Sanders type 4, 6 open fractures). A total of 13 patients (8.39%) sustained spinal fractures.

### 2.2. Time Management

The general time period from trauma to primary diagnosis was within 24 h in 152 cases. One patient was presented to the hospital 4 days after the injury, another fracture was not identified until 7 days later due to the absence of an initial X-ray, and a third was recognized 10 days post-injury due to a misdiagnosed primary X-ray examination.

Of the 152 patients, 135 were admitted to our facility within 24 h of the trauma, and 16 within one week. The remaining 4 patients came between days 10 and 19 after the trauma.

Following admission, 43 fractures were surgically treated on the same day, 74 the following day, 30 between days 2 and 3, and an additional 16 had surgical intervention between days 4 and 7. The decision to delay surgery for five patients by 10 days was due to life-threatening injuries in three cases, all within the context of polytrauma. In one case involving an 80-year-old patient, the treatment plan was switched from conservative management to surgery 13 days after the injury. Another patient underwent surgery on the 11th day due to an initial incorrect diagnosis.

Patients without additional injuries or comorbidities requiring ongoing treatment were discharged an average of 4.77 days after surgery, while the overall average length of stay was 8.56 days (range: 1 to 90 days).

The number of surgeons performing the surgical procedures varied over time. On the day of admission, 12 surgeons were involved; on day 1, 9 surgeons; on days 2 to 3 and 4 to 7, 5 surgeons. Later interventions were carried out by only one surgeon.

**Figure 1:**
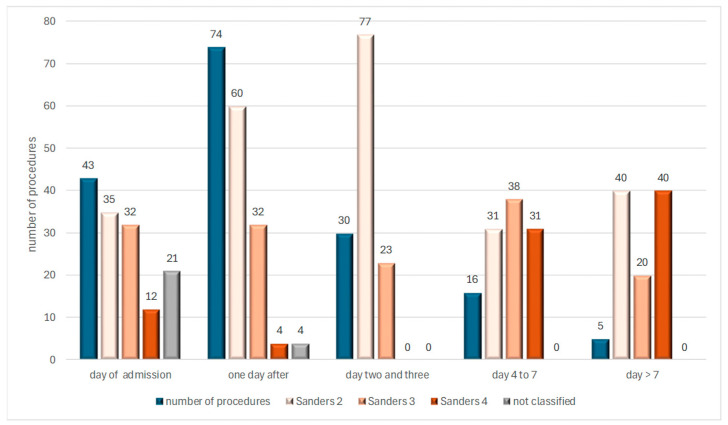
An overview of the timing of surgical procedures according to the Sanders classification.

In Figure 1 the first column presents the absolute number of procedures on the day of admission, 1 to 2 days after admission, 4 to 7 days after admission, and later. The other 4 columns show the percentage distribution of Sanders groups 2, 3, and 4, as well as the unclassifiable fractures (Figure 1).

### 2.3. Statistical Analysis

All data were analyzed using descriptive and inferential statistical methods to evaluate the outcomes across the surgical timing groups. Descriptive statistics, including the means, medians, and standard deviations, were calculated for key variables, such as the AOFAS and FAOS scores. Outlier data points were adjusted to minimize their influence on the analysis.

To compare outcomes across the surgical timing groups, descriptive comparisons were used to observe trends. While statistical tests for significance were not applied to the surgical timing groups due to the small sample size in some groups, the descriptive data showed no clear trend, indicating a significant impact of surgical timing on functional outcomes.

The Sanders severity scoring system was applied to ensure that differences in fracture severity were accounted for when analyzing the outcomes. This scoring system enabled us to stratify patients by fracture complexity and assess whether severity influenced the observed outcomes.

Given the limited sample size in certain timing categories, we acknowledge that the study may not have sufficient power to detect subtle differences among groups.

### 2.4. Method

All patients were treated according to a comprehensive minimally invasive protocol with minor adjustments based on the individual surgeon’s approach. The protocol encompasses standards for fracture analysis, classification, positioning, image-guided technology, surgical technique, osteosynthesis, physiotherapy, and follow-up care. This approach has evolved empirically since 2007. Due to the low complication rates observed in our previous studies [26], surgical treatment was performed on nearly all patients, regardless of age, comorbidities, or fracture type.

Surgical approaches consisted of stab incisions for 144 procedures, the sinus tarsi approach for 16, a medial incision for 1, and various other approaches for 9 procedures, primarily in cases of open fractures.

Fracture reduction was frequently facilitated by a specialized distraction tool (Froehlich distractor [27,28]) in 100 cases. The Westhues technique [29,30,31] was used in 25 cases, and the remaining fractures were reduced using raspatories, bone tampers, and pins.

To stabilize the length and axes, we used fully threaded screws with a diameter of 7.2 mm in 144 cases, usually using 2 of these screws (118 fractures). For sustentaculum, tuber, or processus anterior screws, we used 4.00 mm lag screws. (137 fractures). In 5 cases, K-wires were used alone, all of which were open fractures, and in 10 additional cases, the screws were combined with K-wires. In 1 case, we used an external fixation, and 3 cases of open fractures were treated only with surgical debridement.

**Figure 2:**
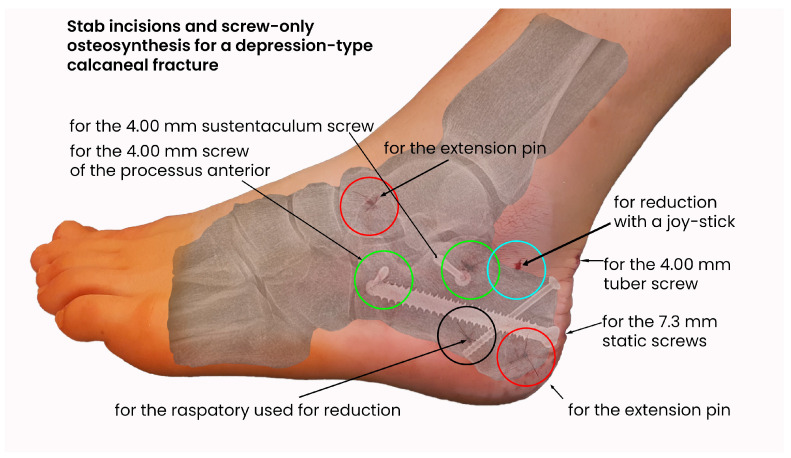
Example of a surgical approach using stab incisions and screw-only osteosynthesis.

The next picture shows the stab incisions for the distractor (red), for the reduction maneuvers with a raspatory (black) and a pin (blue), and for the lateral (green) and dorsal placements of the screws. The radiological outcome is presented as an overlaid X-ray (Figure 2).

For the procedures, 7.3 mm full-threaded screws, 4.0 mm lag screws, and K-wires were used. The overall number of procedures was 168 (excluding the 2 reoperations). Table 2 shows the number of specific screws and K-wires used for several fractures. For example, we used two 7.3 mm screws in 118 cases (row 3, column 2). We did not use any screws for 9 fractures (row 1, column 4) (Table 2).

The average surgical time was 76.2 m. The durations ranged from less than 30 m for 14 procedures to around 4 h for 2 cases.

Only 13 fractures were treated between 10 p.m. and 6 a.m.

**Figure 3:**
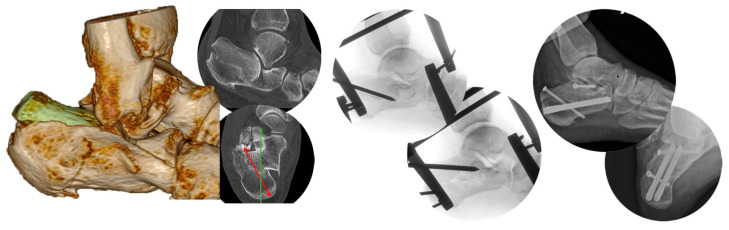
Example of a tongue-type fracture with varus and shortening of the dorso-plantar fragment. Sanders’s classification group 2. (Red arrow shows the angular shift into the varus deviation; Green arrow shows the axis of the calcaneus).

In the following example of a tongue-type fracture (Sanders group 2) the first surgical step was to correct the varus and then address the shortening of the dorso-lateral fragment through distraction. We always referred to the axial view on the CT examination, as shortening cannot be reliably assessed from the lateral view alone. In the central part of the image, we demonstrate the reduction of the tongue fragment using a Schanz screw inserted dorsally. On the right side, we present postoperative X-rays. Immediate fixation-free functional treatment was initiated (Figure 3).

**Figure 4:**
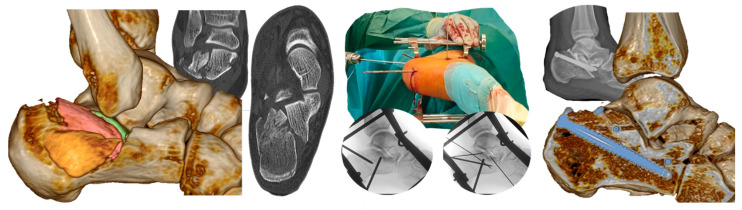
Example of a depression-type fracture with a lateral joint fragment, a central joint fragment, a “blow-out” fragment, shortening, slight varus, and fracture of the processus anterior calcanei. Sanders’s classification group 3.

Next example presents a depression-type fracture (Sanders group 3). Again, we started with correcting the varus and shortening through distraction. Then, we lifted the dorso-lateral fragment dorsally with a pin, reduced the central fragment from the plantar side with a bone tamp, addressed the processus anterior, and inserted the guidewires for the screws. The surgical situation and examples of X-rays are shown in the center. On the right side, we present a radiological control image taken after surgery and a CT control taken 3 months later (Figure 4).

**Figure 5:**
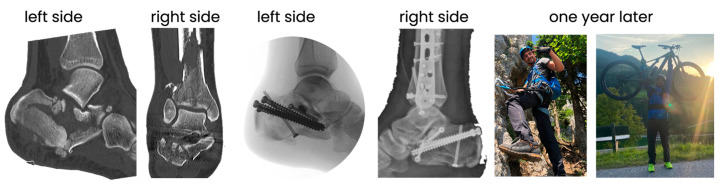
Example of comminuted fractures in a case with fractures on both sides caused by a fall from height. On the right side, there was an additional a pilon fracture. Sanders’s classification group 4.

The last case is a patient with comminuted fractures on both sides combined with a lower leg fracture. The pilon fracture on the right side was reduced and stabilized using the standard open technique. The calcaneal fractures were treated percutaneously with stab incisions and screw-only osteosynthesis. The main goal was to restore height, length, and axes, while ensuring stable osteosynthesis. We also tried to achieve the most anatomical reduction possible of the subtalar joint area. We never performed a primary arthrodesis and opted for fixation-free aftertreatment. A very good clinical result was achieved, supported by the highly motivated and athletic patient (Figure 5).

### 2.5. Postoperative Care

A total of 99 patients were immobilized postoperatively with a plaster cast. Due to the increasing experience regarding the stability of screw osteosynthesis, there has been a significant trend toward fixation-free early functional treatment.

**Figure 6:**
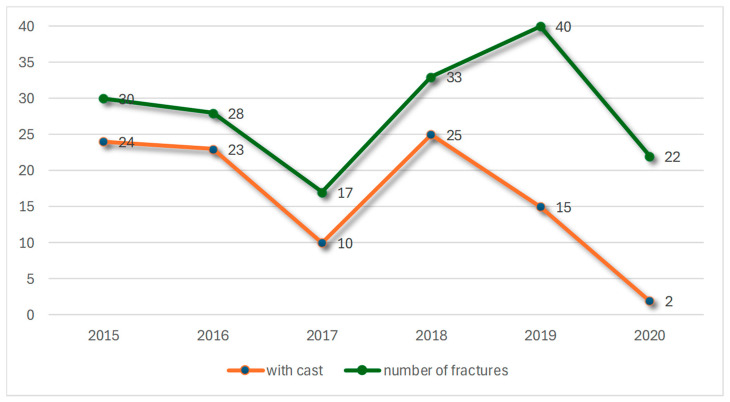
Frequency of cast fixation in the years 2015 to 2020.

This overview shows the number of fractures per year (green) and the decreasing number of fractures treated with a cast (orange) (Figure 6).

The durations of immobilization varied: 13 patients were immobilized for 3 weeks, 81 patients for up to 6 weeks, and 8 patients for longer than 6 weeks, with an average immobilization period of 37.28 days. While most of the surgeons used plaster casts in 83.33% of cases, the surgeon with the most experience used it in only 38.28% for postoperative treatment. After 2019, the senior surgeon never used postoperative fixation and instead started early functional therapy the day after surgery.

A mean of 74.2% of patients began partial weight-bearing at 6 weeks. Full weight-bearing was achieved after 6 weeks in 15 cases, and 147 fractures (90.74% of 162 fractures, excluding 6 cases with no follow-up documentation) were fully weight-bearing by 12 weeks.

In order to ensure safe follow-up treatment, especially in the sensitive phase when progressing to full weight-bearing, CT scans were frequently used. This provided valuable information about bony healing, the exact position of the screws, and any secondary changes in the reduction results.

A total of 6 patients were discharged without reappointment because they went back to their home countries. Follow-up treatments were appointed for all other patients. For 90 patients, the treatment was finished within the first year, and for a further 35, within the second year.

The other patients still had check-ups and continued controls for 5 years after surgery.

**Figure 7:**
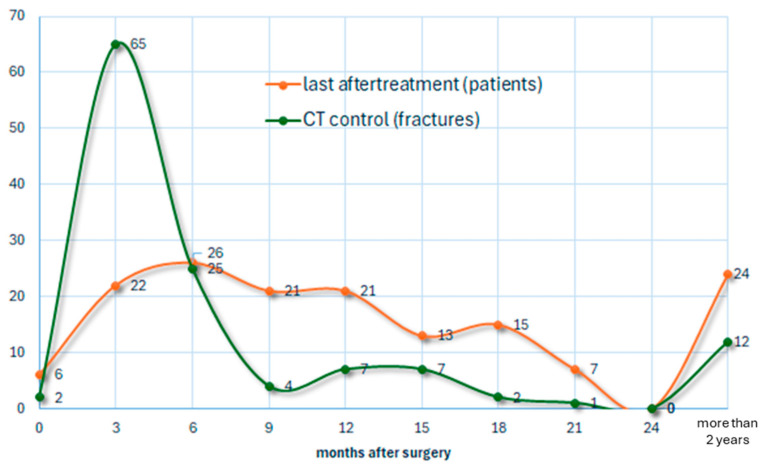
CT controls and aftertreatment.

The chart shows the last appointments in our postoperative care (orange), as well as timing and frequency of the CT controls (green). Excluding 6 cases with no follow-up documentation, all patients had X-ray controls, and 125 fracture patients had a CT control (Figure 7).

## 3. Results

There is no generally accepted scheme for evaluating the outcome and success of surgically treated calcaneal fractures. Commonly used parameters for assessment include the Boehler’s angle, subtalar step-offs or gaps, as well as the length, width, axis, and height of the calcaneus. From our understanding, only a CT examination is meaningful for the radiological results.

### 3.1. Boehler’s Angle

The Boehler’s angle improved from a mean of 8.52 degrees preoperatively to 25.00 degrees postoperatively.

**Figure 8:**
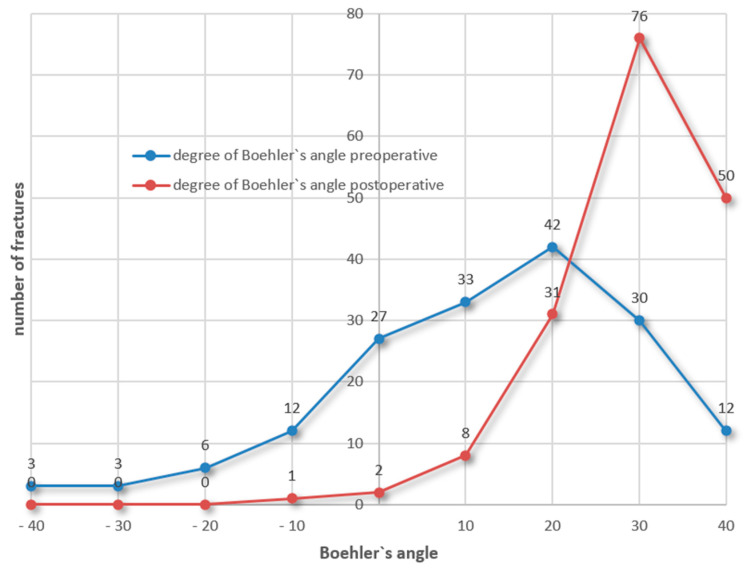
Boehler’s angle.

The figure shows the changes in the Boehler’s angle from the preoperative situation (blue line) to the postoperative results (red line). The x-axis indicates the spectrum of the observed pre- and postoperative Boehler’s angles as degrees. The y-axis depicts the number of fractures (Figure 8).

### 3.2. Infections

A total of three superficial infections (1.76%) were detected. One patient, who had an open fracture, was treated surgically on the day of admission, while the other two patients underwent surgery the following day; one of them had a dislocation of a K-wire. All infections resolved without long-term complications or consequences.

Deep infections occurred in three cases (1.76%), including two open fractures (Sanders Type 4) and one case of direct trauma with open defects in the calcaneus.

Two of these patients received immediate treatment, while the third was treated the following day.

One of these patients developed chronic osteomyelitis with initial intermittent drainage but minimal symptoms. After five years, no further fistula formation was observed, and the patient declined additional surgical intervention. The second patient, an 80-year-old woman with a local defect after direct trauma, developed a deep infection and required partial amputation of the calcaneal tuberosity. The third patient, who sustained Sanders Type 4 fractures bilaterally following a suicide attempt, was also undergoing chemotherapy for cancer. This patient was treated with antibiotics alone without the need for surgical intervention.

According to the data in the literature, the infection rate using the ELA ranges from 13.3% to 31.2%, and the STA shows an overall percentage of infection of 3.6–6.3% [32].

### 3.3. Secondary Dislocations

Secondary loss of reduction was observed in five cases.

One patient sustained an open fracture that was stabilized exclusively using drill wires. Because of an infection one week after surgery, the pins had to be removed, and we saw a dislocation of the fragments. In the remaining four cases, technical filures were identified, including screws placed in the fracture gap and, in one case, intra-articular placement.

Three of these patients underwent surgery on the day of admission, one on the following day, and one after six days due to a polytrauma situation.

### 3.4. Need for Secondary Operation

The need for an early secondary operation was detected in three patients. In one case, a planned reoperation was performed four days after the initial K-wire fixation in a polytraumatized patient in order to facilitate early functional treatment. In another case, a 7.2 mm screw was replaced two weeks postoperatively due to perforation of the medial calcaneal wall.

Another early unplanned removal of K-wires occurred in one patient with deep infection.

One pin was removed without anesthesia 4 weeks postoperatively due to a dislocation and perforation of the skin. In cases where K-wires were used, removal was planned after bony healing—typically between the 7th and 12th week.

The removal of 4.00 mm sustentaculum screws is avoided as much as possible, as there is a risk of injuring the sural nerve due to local scarring.

The removal of larger screws (7.2 mm) is technically straightforward and can be performed under local anesthesia, if requested by the patient. Occasionally, patients report unsightly scars or pressure from footwear near the screw heads. In 25 cases, these screws were removed after a mean of 6.8 months (range: 3 to 15 months). Some patients requested screw removal despite no complaints. A total of another 30 cases underwent screw removal after a mean duration of 11.88 months (range: 4 to 18 months).

### 3.5. Follow-Up and Scores

The functional outcomes, as measured by AOFAS and FAOS scores, were analyzed across different Sander’s fracture classifications to assess the impact of fracture severity.

We successfully followed up with 73 patients who had a total of 79 fractures (6 patients had bilateral fractures). The mean follow-up period was 4 years and 3 months (range: 1 year 11 months to 7 years 6 months). The mean age of the patients was 55.79 years at the time of surgery.

A total of 40 patients (with 44 fractures) sustained additional injuries, and 30 patients had fractures involving the same foot.

The mean AOFAS score for all cases was 91.08, while the mean FAOS was 88.66.

Patients with Sanders type 2 fractures achieved the highest average AOFAS scores (mean: 93.83) and FAOS scores (mean: 91.84), followed by those with Sanders type 3 (mean: 89/84.43) and type 4 fractures (mean: 78/79.66).

The next table shows an overview of the results of the scores obtained, divided into the main groups and subgroups. AOFAS = American Foot and Ankle Society Score; FAOS = Foot and Ankle Outcome Score; number = number of fractures corresponding to each category; max. = maximum; min. = minimum; Sanders = classified calcaneal fractures according to the Sanders classification (Table 3).

### 3.6. Timing of Surgery and Scores

When analyzing with statistical methods (Winsorizing), the AOFAS/FAOS scores in relation to the timing of surgery are as follows: 87.3/84.5 for the patients treated immediately, 91.4/90.2 for those treated the following day, 93.5/90.6 for those treated on days 2 and 3, 93.4/89.8 for those treated between days 4 and 7, and five cases with scores of 89.8/83.5 for surgical treatment after 7 days (see Table 4).

Notably, the group treated on the day of admission included nine patients with open fractures, and a high proportion of fractures not classified in the Sanders system, including four “duckbill fractures”, requiring urgent surgery.

To better correlate the scores with the severity of the fractures in each group, a scoring system was developed by the senior surgeon. Sanders type 2 fractures received one point, type 3 fractures received two points, and type 4 fractures received three points. Fractures that could not be classified within the Sanders system were assigned one point. An additional point was awarded for open fractures. The total score was then divided by the number of fractures in the group. We call this scoring system the “Sanders severity score”.

Table 4 shows all fractures for which an AOFAS/FAOS score could be determined in relation to the time of surgery after admission to show whether the timing of surgery had an influence on the clinical outcome. In order to compare the mean severity of fractures per group, we defined a (Sanders) severity score, with lower values indicating lighter mean fracture patterns and higher values indicating greater severity. The mean surgery time and mean Boehler’s improvement was also added. The day of admission = surgery on the same day as admission; one day = surgery in the first 24 h; 2–3 days = surgery after 2–3 days; 4–7 days = surgery between days 4 and 7; >7 days = surgery after 7 days; AOFAS = American Foot AND Ankle Society Score; FAOS = Foot and Ankle Outcome Score; Sanders = classified calcaneal fractures according to the Sanders classification; SD = standard deviation; n.a. = not applicable (Table 4).

In order to apply the parametric analysis methods provided for this purpose, the AOFAS and FAOS were first checked for outliers using box plots. The identified outliers were limited to a minimum value of the 5th percentile or a maximum value of the 95th percentile using Winsorizing.

Source: Nembhard, H. B. (2004). Applying Contemporary Statistical Techniques.

## 4. Discussion

### 4.1. Timing of Surgery

The timing of surgery for calcaneal fractures remains controversial and depends on various surgical techniques, like the ELA and a wide variety of different methods of MIS, including STA.

Surgery using the ELA is usually not performed within the first 3 days after injury due to concerns about soft-tissue complications.

Delaying surgery until adequate soft-tissue conditions are established was recommended by Sanders (1992, 2014) [20,33]. Al-Mudhaffar (2014) [6] observed an increased risk of complications when surgery was performed within 7 days of injury. Kai Wu (2014) [34] found a 5.47% higher risk of incision complications when surgery was conducted within 3 days, recommending a delay of at least 7 days. Similarly, Zhang (2015) [35] noted in a meta-analysis that surgery should not be performed within the first 3 days post-injury. De Boer (2018) [36] also reported a higher risk of complications for patients who underwent surgery within 2 days of trauma.

To define the ideal timing for surgery, several authors, including Sanders (1992), Attinger (2001), Shuler 2001, Wei Zhang (2015), and Spierings (2020) [20,32,37,38] have recommended waiting for the appearance of the “wrinkle sign”, which can be observed typically between days 7 and 14 after injury.

Regarding the issue of how long one can wait to carry out the procedure, these authors advise against delaying surgery beyond 3 weeks. This recommendation is supported by Tennent (2001) [39], who observed an increased complication rate when surgery was delayed beyond 14 days, and Kwon (2015) [40], who found that delayed operative fixation did not reduce complication rates. Similarly, Jun SU (2017) [41] reported a higher incidence of infections in patients who underwent surgery after an average of 14.2 days, compared to those who were surgically treated after a mean of 11.2 days. Lei Shen (2022) [42] and Spierings (2020) [36] also found that complications increased when surgery was delayed beyond 11 days.

However, there are studies with differing conclusions. Backes (2013) [8] found no significant impact of surgical timing on outcomes. Ho (2013) [7] compared patients operated on within 3 days, after 3 to 10 days, and after more than 10 days and found no statistical differences in infection rates, suggesting that early surgery can be beneficial. Similarly, De Boer (2018) [35] found no significant difference in complications between surgeries performed within 3 to 7 days and those delayed for 8 days or more.

In summary, most surgeries using the ELA are performed within 5 to 14 days after injury, as noted by Sanders (1992), Howard (2003), Backes (2013), Kai Wu (2014), Wei Zhang (2015), Amirul Islam (2020), Spierings (2020), LaRose (2022), and Lei Shen (2022) [20,33,34,36,42,43,44,45,46].

To minimize soft-tissue complications, there is a growing trend toward the use of less invasive techniques, particularly the use of the sinus tarsi approach (STA). Its ability to provide excellent visualization of the lateral joint area, along with a much smaller incision, has contributed to a significant reduction in infection and complication rates, as demonstrated by several studies comparing the STA with the ELA (Basile, 2023; Schepers, 2017; Mehta, 2018; Bandyopadhyay, 2023) [12,15,21,47,48].

Due to the lower surgical risk associated with the STA, there has also been a clear shift toward earlier surgical intervention.

Basile (2023) [21] reported significantly shorter waiting times for the STA, and this finding was also supported by Mehta (2018) [15] in his meta-analysis. Schepers (2017) [12] found that surgery in the STA group occurred a median of 4 days earlier, with shorter postoperative hospital stays. Amirul Islam (2020) [45] reported an average waiting time of 6.6 days for the STA compared to 9.8 days for open reduction and internal fixation (ORIF). Similar results have been observed by LaRose (2022), Bandyopadhyay (2022), and Syros Alina (2022) [46,48,49].

In addition to a lower complication risk, there are technical reasons that make earlier treatment appear preferable. Swords 2016 [50] mentioned that small incisions or sinus tarsi approaches can be used for many calcaneus fractures, but reduction is less difficult when performed early. The smaller incision provides a more limited view of the entire fracture, making manipulation of the fragments more challenging as adhesions develop and bony healing begins, particularly compared to the ELA.

Kwon (2015) [40] and Syros Alina (2022) [49] found that the time to surgery was the only significant predictor of postoperative complications, with a higher rate of complications observed in surgeries performed after 14 days.

### 4.2. Screw-Only or Plate Osteosynthesis

For the sinus tarsi approach (STA), surgeons cannot use the large plates typically associated with open surgery techniques, like the ELA. Instead, specialized smaller plates are provided by companies. However, it is necessary to release the peroneal tendon sheaths and surrounding soft tissues to ensure proper placement of the hardware.

As an alternative to plates, fractures can also be stabilized using a screw-only technique.

A biomechanical study by Smerek (2008) [51] demonstrated that percutaneous screw fixation for Sanders type 2B calcaneus fractures provided strength comparable to that of plating. DeWall (2010) [18] noted that the average loss of reduction at healing was not significantly different between screws and plates. Baoyou (2016) [52] showed in his meta-analysis that cannulated screw fixation and plate fixation offered similar effectiveness in fracture stabilization. Ivanov and Rodemund (2022) [53] tested various screw configurations, all of which demonstrated good stability. Luo Gang (2022) [54] also confirmed the stability of percutaneous screw fixation.

Some authors, such as Sato (2023) [55], reported less loss of Boehler’s angle in the plate group, while Weng Q (2020) [30] noted enhanced stability with plates. However, both studies found comparable clinical outcomes between screw and plate fixation. Lappalainen (2024) [56] indicated that fracture reduction remained consistent with ORIF.

Despite ongoing debates regarding stability, all studies have reported comparable functional outcomes and scores for plating versus screw fixation. Driessen (2021, 2022) [57] and Zhiguo Chen (2022) [54] found no significant differences in the AOFAS scores between the two techniques. Many authors, including Tornetta (2000), Baoyou (2016), van Hoeve (2016), Hegde (2016), Gaul (2018), Philip (2019), Rodemund (2020), Wang Q (2021), Steelman (2021), and Ebrahimpour (2021), [9,23,52,58,59,60,61,62,63,64] mostly recommend screw-only osteosynthesis for all types of calcaneal fractures, especially for tongue-type fractures, due to its advantages of reduction quality, time efficiency, and reduced risk of soft-tissue complications.

Our findings demonstrate that screw-only osteosynthesis provides sufficient stability for the management of intraarticular calcaneal fractures, as reflected by the favorable functional outcomes and low overall complication rates. However, we acknowledge that secondary dislocations occurred in five cases, and this warrants further discussion.

A detailed analysis of these cases indicates that secondary dislocations were primarily due to technical factors, such as improper screw placement.

To minimize such risks, meticulous attention to surgical technique is crucial. This includes precise preoperative planning and the use of intraoperative imaging modalities, such as fluoroscopy or intraoperative CT, to verify reduction quality and screw positioning. Additionally, continued education and experience with minimally invasive techniques can further reduce the incidence of technical errors.

### 4.3. Percutaneous Reduction

Combining screw-only fixation and percutaneous reduction promises a very low risk of postoperative soft-tissue problems, such as infection or wound edge necrosis.

The practice of reducing fractures without open surgery has a long history, with reports dating back to 1855 (Clark, [65]) describing distraction techniques. A significant advancement in fracture treatment came with the invention of X-rays in 1895, which allowed fractures to be analyzed without direct visualization. C.W. Goff (1938) [66] provided a comprehensive overview of 43 different methods dating back to 1906, some of which involved covered techniques.

P. Essex-Lopresti (1951) [25] made significant advancements in the treatment of calcaneal fractures by developing a comprehensive understanding of fracture patterns, classifying them into tongue-type and depression-type fractures. He also described a standardized percutaneous reduction technique for tongue-type fractures based on the concepts of Westhues (1934) [67], which remains highly effective today.

The development of precise CT imaging, including 3D reconstruction, has been crucial for the thorough analysis of complex fractures and for meticulous surgical planning (Lu et al., 2024; Misselyn, 2021; Galluzo, 2018 [68,69,70]). Modern intraoperative 3D scanning now facilitates real-time evaluation and immediate correction during the surgical process (Gwak, H.C., 2015 [71]).

Reduction without a direct view of the fracture is difficult but can be supported by specialized tools. Most calcaneal fractures require the correction of axes and length, and reduction can be supported following the principle of ligamentotaxis (Schepers 2009) [72].

Some surgeons have employed single-point techniques, such as those described by Stulik (2006) [73] and Swords (2017) [50], while others have used specialized tools, such as the two-point distractor designed by Peter Froehlich (1999) [28], as seen in studies by Mattiassich (2017), Rodemund (2020), Batıbay (2020), Harrasser (2023), Li Xu-song (2023), and Froehlich (1999) [23,26,28,74,75,76]. Additionally, the three-point distraction system, described by Forgon and Zadravecz in 1983, was further explored by Driessen (2022) and Schepers (2007) [57,77].

The percutaneous technique also influences surgical timing, with studies indicating that the quality of fracture reduction improves significantly when surgery is performed earlier (Chaniotakis 2023) [78]. In the study of Peng Ye (2019) [27], operations in the closed reduction group were conducted within 1–3 days. Mattiassich and colleagues [26] presented 212 fractures, of which 168 (79.2%) were surgically treated within the first day.

In our patient cohort, we predominantly employed stab incisions (144 cases) for fracture reduction, followed by screw-only osteosynthesis. With proper fracture reduction and careful, accurate screw placement, we consistently achieved stability during the postoperative period to avoid secondary dislocations and enable bony healing. Although the earliest possible time of surgery is usually recommended for percutaneous techniques, our results show that comparable results can also be achieved within the first 7 days after admission. It is essential that all screws are correctly positioned, as demonstrated by four cases of secondary dislocation. To ensure optimal screw placement and assess the reduction, intraoperative CT imaging is recommended.

### 4.4. Additional Factors for Timing

In addition to medical indications for urgent surgery, such as certain fracture types (e.g., duckbill fractures) or open fractures, the timing of surgical intervention depends on various other factors. These include the presence of additional injuries, ranging from frequent associated injuries of the spine or pelvis to polytrauma situations, which may necessitate delaying treatment of the calcaneal fractures to a later stage. Other factors include comorbid conditions or anesthesiologic concerns.

The circumstances of the treating hospital also play a significant role. It is often not feasible to mobilize the necessary personnel and organizational resources for a highly specialized procedure within a short timeframe. Ultimately, a key consideration is whether to proceed with early surgery or delay the intervention when a highly specialized foot and ankle surgeon is not immediately available. Although there was no statistical significance, according to Table 4, it can be seen that urgent and more severe fractures were treated on the day of admission, “easier” cases were treated on days 1 to 3, and “more severe” fractures were treated later. Several different surgeons were involved in the first few days. This number decreased after a few days, as it became easier to book specialists in foot and ankle surgery (day-of-admission procedures employed 12 different surgeons, procedures on days 4 to 7 employed five different surgeons). In this context, we consider it very important that our results on the timing of surgery within 7 days show no significant differences in clinical outcomes.

## 5. Limitations

A major limitation of this study is that we could not include plate osteosynthesis as a comparable group to the screw-only or K-wire techniques.

We began in 2007 with the concept of minimally invasive therapy, inspired by the work of Prof. Peter Froehlich from Hungary. Since then, we have incorporated various methods and standardizations for positioning, X-ray views, reduction techniques, osteosynthesis, and aftercare, all of which have been developed through cadaver workshops, literature reviews, and the thorough documentation of all cases. Given our experience and low infection rates, we chose not to alter our approach for the potential requirements of a study, as doing so would have increased the treatment risk for patients under our care.

For the same reason, we did not have a randomized control group regarding the timing of the surgery. Depending on the medical and organizational factors, surgery was always performed as soon as possible.

Another limitation is the small number of patients available for the AOFAS/FAOS assessment. Our hospital serves as a regional trauma center in an industrial town, treating many patients referred from other hospitals and numerous industrial workers from abroad. Additionally, polytrauma patients, in particular, were difficult to include, which is likely to have lowered the overall AOFAS/FAOS scores. Consequently, the statistical results regarding the timing of surgery and outcomes are limited, and we hope that future studies will provide more precise verification or supplementation of these findings.

Regarding personnel resources, it is also important to note that the follow-up examinations were conducted by a single surgeon, which introduces the potential risk of subjective bias in the results.

We intentionally included all calcaneal fractures treated between 2015 and 2020 in the study, without excluding any specific fracture types. This makes the group incongruent to a certain extent, but also includes all requirements for the treatment of this type of fracture. This study included both closed and open fractures, as well as peripheral, intra-articular, and special types such as “duckbill fractures”. Our goal is to develop a general, minimally invasive concept that applies to all fracture patterns, with standardized fracture analysis, therapy-related classification, and treatment protocols.

To compare the “timing” groups regarding the different severities of fractures within the groups, we designed an individual, unofficial scoring system and called it the “Sanders severity score”.

This study focused on two main topics: the timing of surgery and screw-only osteosynthesis. Many other factors influencing complication rates and outcomes, like fracture severity, surgical techniques, age, aftercare etc., were not analyzed in depth or individually. Including all of these issues would have expanded the extent of the study and detracted from the clarity of its main focus. We will consider them in further studies.

## 6. Conclusions

The results of this study, along with the existing literature, demonstrate that minimally invasive techniques promise good outcomes in the surgical treatment of calcaneal fractures. Percutaneous techniques, which minimize additional iatrogenic soft-tissue trauma, especially lead to very low complication rates. This approach allows for early surgical intervention, not only in cases of severe swelling or beginning blister formations but also in patients with comorbidities, advanced age, or heavy smoking habits. With correct positioning and dimensions, screw-only osteosynthesis provides sufficient stability to facilitate early functional postoperative care and ensure safe bony healing without secondary dislocations. This method also allows for much easier hardware removal in comparison to plate use if necessary. The timing of surgery using these techniques shows no significant differences in outcomes within the analyzed time period of 7 days.

## Figures and Tables

**Table 1:** Overview of patient’s epidemiology, fracture types, and surgical approach.

Patients	all patients	155	male	121 (78.1%)	female	34 (21.95)	right side	85(54.8%)	left side	83 (53.5%)	open fractures	15 (8.92%)
Age	13–20 years	8 (5.16%)	21–40 years	46 (29.68%)	41–60 years	73 (47.10%)	61–80 years	25 (16.13%)	over 80 years	3 (1.94%)		
Fractures	all fractures	168	intraarticular	159 (94.6%)	duckbill	4 (2.38%)	direct trauma	4 (2.38%)	isolate Proc. Ant.	1 (0.59%)	Fx both sides	13 (7.73)
Job	worker	52 (33.54%)	employee	41 (26.45%)	retired	29 (18.70%)	unemployed	17 (10.96%)	self-employed	13 (8.38%)	school	3 (1.93%)
Accident scene	home/private	82 (52.90)	work	52 (33.54%)	sports	11 (7.09)	traffic	5 (3.22%)	sucide	5 (3.22%)		
Accident type	low height	32	1–2 m	54	over 2 m	58	Pinching	11				
Average age	low height	49.5	1–2 m	52.4	over 2 m	41.9	Pinching	38.8	workers compensation	103 (66.45%)	no workers compensation	52 (33.54%)
Additional diseases	diabetes	6 (3.18%)	hypertension	18 (2.26%)	cardial problems	6 (3.87%)	Drug abuse	5 (3.22%)	Smoker	39 (25.16%)	vascular disease	4 (2.58%)
Procedures	170 (2 reop.)	170	Surgeon A	110 (64.7%)	Surgeon B	15 (8.8%)	Surgeon C	13 (7.6%)	Surgeon D	10 (5.9%)	surgeon others	22 (12.9%)
Sanders	group 2	81 (48.21%)	group 3	56 (33.33%)	group 4	17 (10.11%)	not classified	14 (8.33%)				
Sanders subgroup	subgroup 2A	61 (36.30%)	subgroup 2B	15 (8.92%)	subgroup 2C	5 (2.97%)	subgroup 3AB	40 (23.80%)	subgroup 3AC	16 (9.52%)	subgroup 4	17 (10.11%)
Essex-Lopresti	Tongue-type	56 (33.33%)	Depression-Type	87 (51.785)	not classified	25 (14.885)						
Additional fractures	talus	12 (7.14%)	lateral ankle	16 (9.52%)	impingement	16 (9.52%)	navicular bone	3 (1.78%)	processus anterius	93 (55.35%)		
Surgical approach	step-incision	144 (84.70%)	Sinus-Tarsi	16 (9.52%)	medial	1 (0.59%)	other	9 (5.35%)				
Surgical technique	with distractor	96 (56.47%)	distr. & Westhues	4 (2.35%)	Westhues alone	21 (12.35%)	other techniques	46 (27.05%)	no reduction	1 (0.58%)		

**Table 2:** The use of screws and K-wires.

Type of Screws	7.3 mm	4.00 mm	Total number		
Screws/Fracture	Cases	Cases	Cases	K-Wires/Fracture	Cases
no screws	24	31	9	no K-wires	153
1 screw	3	69	4	1 K-wire	6
2 screws	118	52	25	2 K-wires	4
3 screws	21	13	69	3 K-wires	3
4 screws	2	2	40	4 K wires	1
5 screws		1	16	5 K-wires	
6 screws			2	6 K-wires	1
7 screws			2		
8 screws			1		

**Table 3:** AOFAS and FAOS results.

	Fractures	AOFAS			FAOS		
	Number	Mean	Max.	Min.	Mean	Max.	min.
all fractures	79	91.08	100	61	88.66	100	54.01
Sanders Main group							
Sanders 2	43	93.83	100	69	91.84	100	67.84
Sanders 3	28	89	100	61	84.43	100	54.01
Sanders 4	5	78	88	65	79.66	85.87	72.4
not classified	3	93	100	79	97.59	100	92.78
Sander subgroups							
2A	34	92.5	100	69	90.61	100	67.84
2B	7	98.57	100	90	95.46	100	71.31
2C	2	100	100	100	100	100	100
3AB	17	92.23	100	69	88.64	100	55.64
3AC	11	86.54	100	61	80.47	97.85	54.01
3BC	0	0	0	0	0	0	0
4	5	78	88	65	79.66	85.87	72.4

**Table 4:** AOFAS/FAOS scores in relation to timing of surgery after admission.

Surgical Timing After Admission	Overall	Day of Admission	One Day	2–3 Days	4–7 Days	>7 Days	p
Number of fractures	79	16	27	18	13	5	
AOFAS (SD)	91.3 (10.4)	87.3 (11.6)	91.4 (10.5)	93.5 (9.9)	93.4 (9.9)	89.8 (8.8)	0.440
FAOS (SD)	88.7 (13.3)	84.5 (15.4)	90.2 (12.4)	90.6 (13.7)	89.8 (10.9)	83.5 (17.0)	0.538
Sanders (%)							n.a.
not classifiable	3 (3.8)	2 (12.5)	0 (0.0)	1 (5.6)	0 (0.0)	0 (0.0)	
2A	34 (43.0)	5 (31.2)	17 (63.0)	7 (38.9)	4 (30.8)	1 (20.0)	
2B	7 (8.9)	2 (12.5)	2 (7.4)	1 (5.6)	2 (15.4)	0 (0.0)	
2C	2 (2.5)	1 (6.2)	0 (0.0)	0 (0.0)	0 (0.0)	1 (20.0)	
3AB	17 (21.5)	3 (18.8)	3 (11.1)	6 (33.3)	3 (23.1)	2 (40.0)	
3AC	11 (13.9)	2 (12.5)	5 (18.5)	3 (16.7)	1 (7.7)	0 (0.0)	
IV	5 (6.3)	1 (6.2)	0 (0.0)	0 (0.0)	3 (23.1)	1 (20.0)	
Sanders severity score (SD)	1.5 (0.7)	1.6 (0.6)	1.3 (0.5)	1.5 (0.5)	1.9 (1.0)	2.0 (1.2)	0.044
Surgery time (SD)	87.0 (40.0)	101.5 (43.7)	75.2 (29.0)	79.1 (35.8)	112.3 (51.4)	69.2 (31.3)	0.028
Böhler Improvement (SD)	17.1 (12.9)	18.9 (16.6)	16.7 (11.7)	16.3 (9.6)	9.8 (8.1)	34.4 (13.4)	0.007

## Data Availability

The raw data supporting the conclusions of this article will be made available by the authors on request.

## Abbreviations

The following abbreviations are used in this manuscript:

STA	Sinus tarsi approach
ELA	Extended lateral approach
MIS	Minimally invasive surgery
AUVA	Workers Compensation Board Austria
